# Exploration of Data Space Through Trans‐Dimensional Sampling: A Case Study of 4D Seismics

**DOI:** 10.1029/2021JB022343

**Published:** 2021-11-26

**Authors:** Nicola Piana Agostinetti, Maria Kotsi, Alison Malcolm

**Affiliations:** ^1^ ZED Depth Exploration Data GmbH Vienna Austria; ^2^ PanGeo Subsea Inc. St John's NL Canada; ^3^ Earth Sciences Department Memorial University of Newfoundland St John's NL Canada

**Keywords:** data‐space exploration, Bayesian inferences, trans‐D sampler

## Abstract

We present a novel methodology for exploring 4D seismic data in the context of monitoring subsurface resources. Data‐space exploration is a key activity in scientific research, but it has long been overlooked in favor of model‐space investigations. Our methodology performs a data‐space exploration that aims to define structures in the covariance matrix of the observational errors. It is based on Bayesian inferences, where the posterior probability distribution is reconstructed through trans‐dimensional (trans‐D) Markov chain Monte Carlo sampling. The trans‐D approach applied to data‐structures (termed ”partitions”) of the covariance matrix allows the number of partitions to freely vary in a fixed range during the McMC sampling. Due to the trans‐D approach, our methodology retrieves data‐structures that are fully data‐driven and not imposed by the user. We applied our methodology to 4D seismic data, generally used to extract information about the variations in the subsurface. In our study, we make use of real data that we collected in the laboratory, which allows us to simulate different acquisition geometries and different reservoir conditions. Our approach is able to define and discriminate different sources of noise in 4D seismic data, enabling a data‐driven evaluation of the quality (so‐called “repeatability”) of the 4D seismic survey. We find that: (a) trans‐D sampling can be effective in defining data‐driven data‐space structures; (b) our methodology can be used to discriminate between different families of data‐structures created from different noise sources. Coupling our methodology to standard model‐space investigations, we can validate physical hypothesis on the monitored geo‐resources.

## Key Points

- We apply a trans‐dimensional approach to data‐space exploration for defining unknown data‐structures
- Our novel methodology is able to separate data‐volumes that are coherent with a‐priori physical and not‐physical hypotheses
- In case of 4D seismics, the analysis of the full posterior probability distribution of the data‐structures can be used for classifying different sources of 4D signal

## 1 Introduction

In their investigations of the Earth system, geo‐scientists have to deal with two complementary spaces: data space and model space. The *model space* is generally defined as the space of the investigated parameters. For a given parameterization of the system, each point of the model space defines a possible model of the system, represented by a combination of values of the model parameters. To make inferences on the model parameters, we need to take measurements of relevant geo‐observables. The *data space* contains all the possible combinations of such observations (Tarantola, 2005) and the measured data points form a local subset of the data space with its own structure. While there is a vast literature about methodologies for investigating the model space (e.g., Sambridge & Mosegaard, 2002), few attempts have been made at a systematic exploration of the data space. Exploration of the data space is an ordinary activity for geo‐scientists, and includes, for example, data preparation, quality controls (QCs) for data selection and estimation of data errors. Some of those activities, for example the data selection, could have a strong impact on the data space, modifying, for example, the data structure. Generally, such activities rely on the *expert‐opinion* of the geoscientists and are carried out ahead of the main geophysical investigations that are related to the model space.

There are two main reasons for considering a systematic exploration of the data space. First, the ever growing amount of geo‐data available to geo‐scientists needs to be tackled with more automated workflows; expert opinion is generally a time‐consuming process. Second, more interestingly, expert opinion, as a human activity, implies the separation of data into categories (i.e., a discrete number of outputs) rather than a more general continuous evaluation of probability. For example, in data selection activities, the expert can select and, then exclude, part of the data based on their experience, using a two category model (in/out, good/bad). Conversely, a more automated workflow, developed in a statistical framework, can associate a probability value to each data point, avoiding the need to remove any of them from the analysis.

Exploration of the data‐space is generally associated with Machine Learning (ML) techniques (i.e., the so‐called *data mining*). In fact, ML makes use of huge databases to extract common features of the data themselves and to explore potential correlation between such features (e.g., Huang, 2019; Olivier et al., 2018). In particular, clustering algorithms try to separate different data regions (clusters) based on the criterion or objective function to be optimized (e.g., Van Mechelen et al., 2018). The number of clusters is a key parameter the definition of which is a topic of active research (Arbelaitz et al., 2013). It can imposed a‐priori or chosen during or after the data‐analysis, initial work has been done to relax the constraint on the number of clusters (e.g., the DBSCAN algorithm used in Sabor et al., 2021).

In recent years, some studies have reported cases of systematic exploration of the data space, even if such analyses take often a marginal role in the scientific studies themselves. In particular, there are some examples (Bodin, Sambridge, Rawlinson, & Arroucau, 2012; Dettmer & Dosso, 2012; Xiang et al., 2018) where Bayesian inference is applied to a geophysical inverse problem for defining both physical parameters (i.e., investigating the model space) and the errors associated to the data (i.e., exploring the data space), the so‐called *Hierarchical Bayes* approach (Malinverno & Briggs, 2004). In Hierarchical Bayes algorithms, the uncertainties related to the data are assumed to be poorly known and need to be estimated during the process. This approach usually assumes a fixed number of parameters which represent the unknown part of the data space. In most applications of the Hierarchical Bayes approach, the absolute value of the data errors is considered an unknown in the problem that needs to be inferred (Bodin, Sambridge, Rawlinson, & Arroucau, 2012). Sometimes, in cases where the structure of the data errors is known (i.e., we know which data points are measured with more precision with respect to other points), a scaling factor of the data error is used as the unknown (Piana Agostinetti & Malinverno, 2018). In more complex cases, the Hierarchical Bayes approach is adopted to somehow define a function of the data uncertainties, so‐called “data structures” or “states” hereinafter, which include: estimating an auto regressive model of the data errors (i.e., a form of error correlation, Dettmer & Dosso, 2012), and estimating an increasing linear model for the data errors as a function of the geometrical distance between measurement points (e.g., Galetti et al., 2016). In all of these cases however, the number of parameters representing the data structure is fixed a‐priori (usually one or two parameters, rarely more than three). By contrast, Steininger et al. (2013) and Xiang et al. (2018), extend Hierarchical Bayes approach to make inferences on the data space by considering data structures that are represented by a variable number of parameters. Xiang et al. (2018) make use of a transdimensional (trans‐D) sampler (Sambridge et al., 2006, 2013) for sampling models belonging to two different states: in one state, one unknown defines an autoregressive model of the first order for the data errors, that is, assume uncorrelated errors, while in a second state, two unknowns are used to define an autoregressive model of the second order, that is, exponential correlation between data uncertainties. Using this ability to jump from one state to the other, the algorithm is able to indicate the “predominant” auto‐regressive model associated to the data errors. As far as we know, Steininger et al. (2013) and Xiang et al. (2018) are the first applications of a trans‐D algorithm in Geophysics, for sampling different states representing different error models, even if they are limited to a transition between states represented by one and two parameters.

In this study, we move a step forward in the development of algorithms for data space exploration. We make use of a trans‐D sampler for exploring different “states” (represented by a different number of variables), where each state reproduces a partition of the data space (i.e., a data structure). The number of states to be explored is no longer strictly limited (e.g., two states, like in Xiang et al., 2018), and the number of variables representing each state can vary between a user‐defined minimum and maximum. The algorithm is developed in a Bayesian framework, used to define the posterior probability of the data structures. Data space structures are expressed in terms of partitions of the covariance matrix of the errors, which allow us to define regions of the data space where measured data are in agreement with a given working hypothesis. The algorithm is applied to the data analysis workflow used for time‐lapse seismics (also called *4D tile lapse seismics*), a technology used primarily by oil&gas companies for monitoring their reservoirs. The 4D seismic data consist of time‐repeated active seismic surveys that need to be investigated for detecting noise/distortions and focusing the subsequent geophysical inversion on the portion of active seismic data where temporal changes have occurred. The algorithm is applied on laboratory data that mimic active seismic surveys and the results are discussed in light of the potential of the algorithm for statistically separating signals with different origins.

### 1.1 4D Seismics: Key‐Concepts and Present‐Day Challenges

The term *4D seismics* indicates the data workflow adopted by oil&gas companies for monitoring their reservoirs through the repetition, after a few years, of active seismic surveys. The 4D seismic workflow consists of three main phases: acquisition, processing and interpretation. 4D seismics is generally performed for off‐shore reservoirs, but the first successes were obtained on‐shore (e.g., Davis et al., 2003; Porter‐Hirsche & Hirsche, 1998). This technology is also used for monitoring CO_2_ underground storage sites (Cheng et al., 2010; Lumley, 2010; Roach et al., 2015; Yang et al., 2014). Briefly, a first active seismic survey, the so‐called *baseline survey*, is performed just before starting production to image the untouched resources. After some time and while the reservoir is under production, the active seismic survey is repeated, the so‐called *monitor survey*. If the seismic acquisition and data processing are exactly the same as those used for the baseline survey, the differences between the images can be uniquely attribute to changes in the physical properties of the reservoir due to its exploitation. Through the analysis of such differences, scientists can make informed decisions about the next phases of exploitation of the reservoir.

An important question is: how can we get relevant information from 4D seismics? Production related effects on images obtained from the monitor survey can be obscured by distortions induced by the lack of repeatability of the data acquisition and processing. This is one of the main technical barriers for getting the correct information from 4D seismics (Koster et al., 2000). The concept of *repeatability* between two or more seismic surveys indicates the degree to which the data‐sets can be considered to be generated from the same operational and computational workflows. Measures of repeatability between two seismic surveys generally include Normalized Root Mean Square (NRMS) and trace correlation (also called *predictability* Kragh & Christie, 2002). Increasing and evaluating the repeatability of 4D seismics have been the focus of a number of studies in the last decades (Houck, 2007; Landro, 1999; Pevzner et al., 2011), with the main efforts going into increasing acquisition quality, that is, hardware solutions. Statistical approaches to 4D data analysis have been limited to the interpretation phase (e.g., applying Machine Learning algorithms to porosity inversion Dramsch, 2019).

### 1.2 Methodological Framework: Bayesian Inference, Markov Chain Monte Carlo and Trans‐Dimensional Algorithms

Various geophysical inverse problems have been solved following a probabilistic Bayesian framework (Tarantola, 2005, 2006). Bayes' theorem

(1) $$p\left(m|d\right)=\frac{p\left(m\right)p\left(d|m\right)}{p\left(d\right)}$$

connects (probabilistic) prior information *p*(**m**) about some subsurface properties (*m*) and data measured (*d*), generally at the surface, to extract new information about such properties (the so‐called *posterior probability distribution p*(**m**∣**d**) or PPD), through an (assumed) known error statistics (the Likelihood *p*(**d**∣**m**), or *L*(**m**) hereinafter, Bayes, 1763). Thus, in contrast with other approaches, the solution of geophysical inverse problems is given in the form of a probability distribution over the investigated parameters, and not as a single value for each parameter (i.e., a single model). In simple cases, Bayes' theorem can give an analytic solution to geophysical inverse problems (Tarantola, 1987). However, numerical methods have been widely used in more complex cases. In particular, Markov chain Monte Carlo (McMC) sampling has been found to be well suited for sampling a chain of Earth models with a probability proportional to the PPD and, thus, to make inferences on relevant parameters based on such sampled models (Sambridge & Mosegaard, 2002). Here, we follow the approach presented in Mosegaard and Tarantola (1995) and we develop a sampler of the prior probability distribution which can be “switched” to sample models with a probability that follows the PPD. After collecting a relevant number of models from the PPD, we compute numerical estimators of the investigated parameters directly from the sampled models. For example, the mean value of the parameter *m*, can be estimated as

(2) $$\hat{m}=\frac{1}{N_{s}}\sum_{j}^{N_{s}}m^{j},$$

where *N*_*s*_ is the number of samples computed during the McMC sampling and *m* ^*j*^ is the value of parameter *m* for the *j*th model sampled. Following the approach in Mosegaard and Tarantola (1995), we define the probability of accepting a new model along the Markov chain as:

(3) $$\alpha =\min\left[1,\;L\left(m_{cand}\right)/L\left(m_{cur}\right)\right],$$

where **m**_*cand*_, the candidate model, and **m**_*cur*_, the current model, are two consecutive Earth models along the Markov chain and *L*(**m**) is the likelihood of the model given the observed data. In other words, the candidate is always accepted if *L*(**m**_*cand*_) ≥ *L*(**m**_*cur*_). If *L*(**m**_*cand*_) < *L*(**m**_*cur*_), the random walk moves to the candidate model with probability equal to *L*(**m**_*cand*_)/*L*(**m**_*cur*_). The last point, *L*(**m**_*cand*_) < *L*(**m**_*cur*_), guarantees that the McMC sampler will not get stuck in a local maximum of the likelihood function, because models which worsen the fit to the data may still be accepted.

Two fundamental points in Bayesian inferences are the initial states of knowledge about the investigated parameters, the so‐called *priors*, which can take a closed analytical form, or be represented by a set of rules (e.g., one parameter has to be smaller than a second parameter, like in P‐ and S‐ waves velocities in rocks). More interestingly, the statistics of the data uncertainties should be known at a certain level. Such statistics is used to compute the likelihood value of an Earth model. Simplified statistics can be adopted (e.g., a diagonal covariance matrix in Gaussian distributed errors) but has been proven to give un‐realistic results in some cases (Birnie et al., 2020). Both of these assumptions have to hold to make inferences on physical parameters and, given Equation 1, the solution to the geophysical inverse problem may change under different assumptions.

An efficient design of the McMC sampler is fundamental for achieving robust results (in terms of number of samples extracted from the PPD) in a limited amount of time. Several different *recipes* have been designed in the past for proposing a *candidate model*, that is, a new point in the model space, as a perturbation of the *current model*, that is, the last visited point in the model space (Bodin, Sambridge, Tkalcic, et al., 2012). In fact, if the sampling is too limited to the neighborhood of the current model, McMC will converge too slowly toward the global maximum of the likelihood function. Conversely, too strong a perturbation of the current model will likely lead to poorly fitting candidate models, most of which will be rejected. In recent years, one ingredient that has been added to many implementations of the McMC sampler is the possibility of sampling a candidate model which has a different number of variables than the current model (Malinverno, 2002; Sambridge et al., 2006). In practise, we relax the hard constraint of a fixed number of variables in the models, allowing it to vary between fixed minimum and maximum values. This new generation of McMC samplers are collectively called trans‐dimensional samplers (e.g., Sambridge et al., 2013) and are based on the pioneering works of Geyer and Møller (1994) and Green (1995). For trans‐dimensional samplers, Equation 3 holds under specific assumptions on the model space transformation and its Jacobian matrix (see Appendix B in Piana Agostinetti & Malinverno, 2010; for details).

## 2 Data

**Figure 1:**
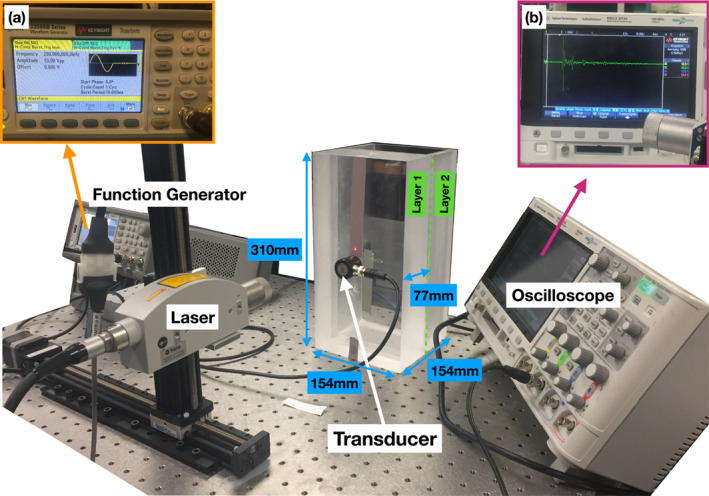
Experimental setup and photos of the equipment. (a) Function generator showing the parameters of the source pulse (b) oscilloscope showing an example of a recorded wiggle. The red spot on the model is the location of the laser receiver, which is moved vertically in controlled increments to generate wiggles at different locations, which are combined into the final shot record.

We consider a simple time‐lapse scenario that consists of an overburden layer and a reservoir. To better mimic a real world application, we use a scaling factor of 10,000 such that a frequency of 200 kHz represents a frequency of 20 Hz, and a dimension of 1 mm represents 10 m. To build this experiment in the lab we take two Plexiglas blocks with dimensions 310 × 154 × 77 mm, and attach them together (Figure 1). The first Plexiglas block represents the overburden layer with elastic properties of *V*_*p*_ = 2,780 m/s, *V*_*s*_ = 1,480 m/s, and *ρ* = 1.19 g/cm^3^. This overburden layer remains unchanged between the two surveys. To build the reservoir layer we remove a rectangular cube from the second block, allowing us to insert different fluids into our “reservoir.”

**Figure 2:**
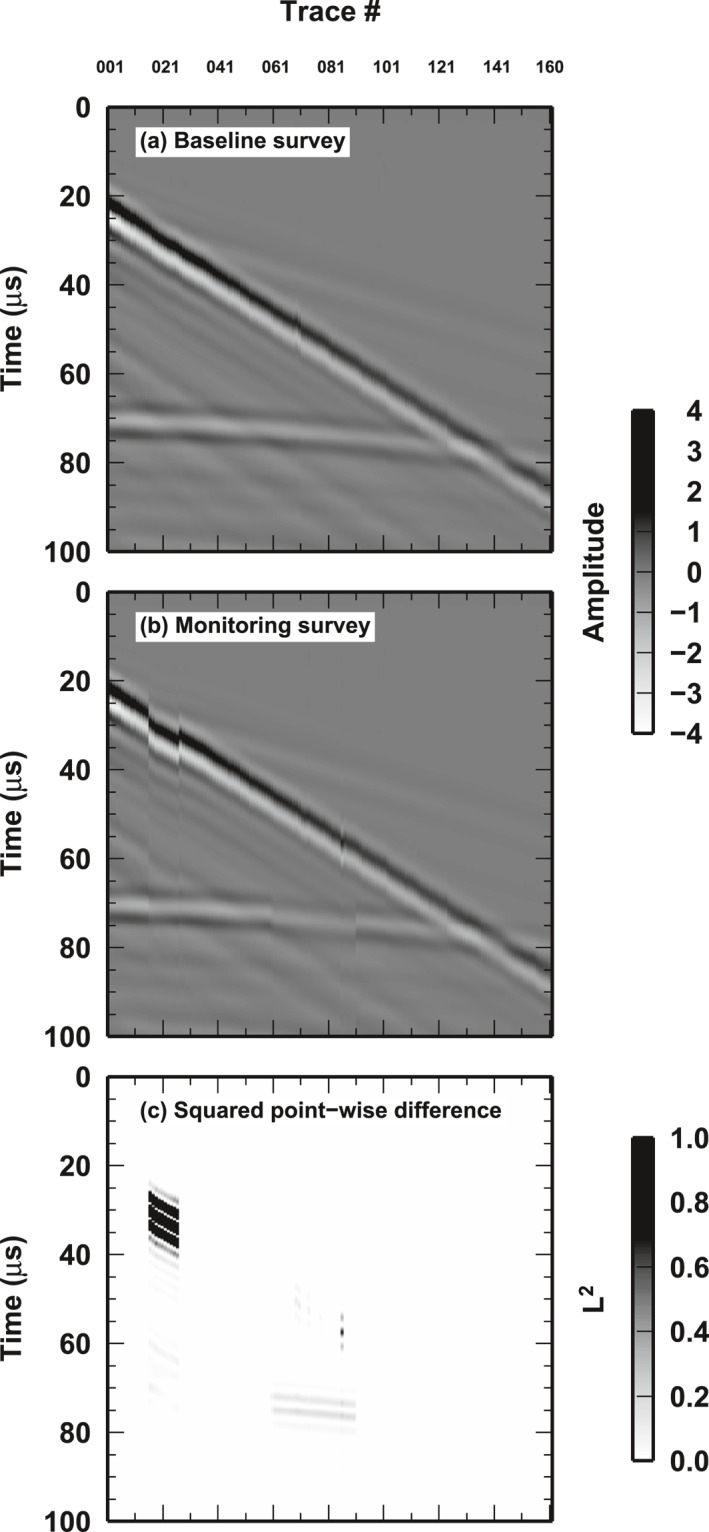
Example of seismic surveys: (a) Baseline survey using all wiggles generated with air/Plexiglas interface. (b) Monitor survey. Same wiggles as in (a), but wiggles from 15 to 25 have been replaced with the wiggles from 19 to 29, same interface (simulating misplaced receivers); wiggles from 60 to 89 have been replaced with wiggles recorded in the same position but with a different interface (water/Plexiglas, simulating a change in the physical properties of the reservoir). (c) Squared differences of the two survey, computed for each sample separately. Notably the largest values are associated with “misplaced receivers.” See Section 4.1 for the details of this experiment.

For the baseline survey, we keep the second block empty, representing a gas‐filled reservoir. In this case, the elastic properties of the air are *V*_*p*_ = 332 m/s, *V*_*s*_ = N/A, and *ρ* ∼ 0 g/cm^3^. For the monitor survey, we fill the block with water, miming a scenario where the gas in the reservoir has been replaced with brine. The elastic properties of the water are *V*_*p*_ = 1,500 m/s, *V*_*s*_ = N/A, *ρ* ∼ 1 g/cm^3^. Figure 1 shows the experimental setup for the data acquisition. For the source, we use a P‐wave transducer with a single‐cycle sine wavelet at 200 kHz, generated through the function generator (top left corner of Figure 1). This P‐wave transducer has a diameter of 10 mm. For the receivers, we use a laser vibrometer that measures the particle velocity along the direction of the laser beam (perpendicular to the surface), and sends it to the oscilloscope to be saved. The laser measures the signal at 160 points along the tape, giving us a total of 160 receivers with a sampling distance of 0.5 mm. The nearest offset in this case is 10 mm. Figure 1 top right corner shows the signal reading at the nearest offset for the baseline case. Throughout the data acquisition the P‐wave transducer is glued to the Plexiglas box, and the laser is attached to a stage that stably moves it along the tape. This allows for a controlled and repeatable time‐lapse experiment. Summarizing, the experimental set‐up allows us to record 160 “wiggles” for each of the two different reservoir‐states, composing two “shot‐gathers.” For the first 100 wiggles in each shot‐gather, clear arrivals from the surface and the reservoir can be separated. These shot‐gathers compose a homogeneous, discrete (*x*, *t*)‐space, where *x* is the wiggle offset, and *t* is the recording time (Figure 2). In order to obtain more copies of the baseline and monitoring surveys without doing the full experiment, we make use of the error model described in Section 2.1, adding a noise component to the original recordings.

In general, we use the first shot‐gather from the first reservoir‐state experiment as the “baseline survey” (Figure 2a). We combine the wiggles for the two experiments to simulate different monitoring scenarios. For example, in Figure 2b, we mimic: (a) the misplacement of some sensors (wiggles between 15 and 25), replacing the correct baseline wiggles with wiggles from the baseline survey but with a four‐wiggles shift; and (b) the presence of changes in the reservoir (wiggles 60 to 90), replacing wiggles from the baseline with wiggles from the second reservoir‐state experiment. In the “monitoring survey” un‐changed wiggles belong to one of the copies of the baseline survey, and not the original baseline data set itself. Point‐wise measurements of the squared difference between baseline and monitor surveys can be larger for misplacement sensors than for reservoir alteration (Figure 2c), making the discrimination between the two effects quite challenging.

To test our methodology, we used one in five wiggles for the first 100 wiggles, thus, we collect 20 “traces” for each survey, *N*_*w*_ = 20. Downsampling the number of wiggles allows us to have enough data for simulating the misplacement of the receiver in the monitor survey. In the following, we continue to call “wiggles” the recording for a single detector position as a function of time in each shot‐gather, and we call “traces” the wiggles selected to compose the baseline and monitor surveys. Each trace is composed of *N*_*S*_ = 1,251 samples. Thus, our (*x*, *t*)‐space is composed of *N*_*w*_ ⋅ *N*_*s*_ = 25,020 data‐points.

### 2.1 Error Statistics

To rigorously compare the monitor and baseline survey we need to know how the errors are statistically distributed in the two data‐sets, that is, the error covariance matrix. Computing the rank of such a large (*N*_*w*_ ⋅ *N*_*s*_) × (*N*_*w*_ ⋅ *N*_*s*_) matrix could be intractable. To avoid this, we estimate the covariance matrix from the data themselves with the following assumptions. First, we do not consider inter‐trace correlation, so our model of the covariance matrix is block‐diagonal, one block for each trace. Note that this assumption means that near‐by traces are not correlated, which could be un‐realistic under some scenarios, for example, weather conditions, acquisition systems and so on. Second, we assume the same error statistics for the baseline and monitor surveys. Again, this assumption could be partially false for, for example, surveys acquired with a large (10s of years) time‐gap. However, under our assumptions, we can estimate a tractable error covariance matrix $C_{\mathrm{e,ij}}^{*}$ which can be decomposed following the approach developed in Malinverno and Briggs (2004), with an adequate correlation model (Kolb & Lekić, 2014) (see Table 1 for variables definition).

**Table 1:** Description of Variables and Terminology

**Variables**	**Description**
*N*_*w*_	Number of traces in the survey
*N*_*s*_	Number of samples per trace
*i*, *k*	Indices for samples
*j*	Index for a trace
**x**_*ij*_	Space (*x*_*i*_) and time (*x*_*j*_) position of the *i*th point for the *j*th trace
*b*_*ij*_	Amplitude of baseline survey at the *i*th point for the *j*th trace
*m*_*ij*_	Amplitude of monitor survey at the *i*th point for the *j*th trace
**e**_**ij**_ = (*b*_*ij*_ − *m*_*ij*_)	Sample‐wise difference between baseline and monitor surveys (at the *i*th point for the *j*th trace)
**Terms**	**Description**
Data	
Shot‐gather	Original data from the laboratory, one for each experimental set‐up
Wiggle	One recording (in time) at a fixed position within one shot‐gather
Survey	Input data for the algorithm: new shot‐gather composed of selected wiggles
Trace	One recording of the survey
4D signal	Differences in the monitoring and baseline surveys
Sources of 4D signal	
Target signal	Changes in reservoir properties
Noise	Ambient random noise
Perturbation	Sensor misplacement

Given the nature of our data, that is, band‐limited waveforms, our covariance matrices are semi‐positive definite Toeplitz matrices and they can be decomposed as:

(4) $$C_{\mathrm{e,ij}}^{*}=SRS$$

where:

(5) $$S=(\begin{array}{ccccc} \sigma_{1,1} & 0 & 0 & \ldots & 0\\ 0 & \sigma_{2,1} & 0 & \ldots & 0\\ 0 & 0 & \sigma_{3,1} & \ldots & 0\\ \vdots & \vdots & \vdots & \vdots & \vdots \\ 0 & 0 & 0 & \ldots & \sigma_{N_{s},N_{w}} \end{array})$$

represents the diagonal matrix containing the standard deviation of each data point *b*_*ij*_ in the baseline (Malinverno & Briggs, 2004).

**Figure 3:**
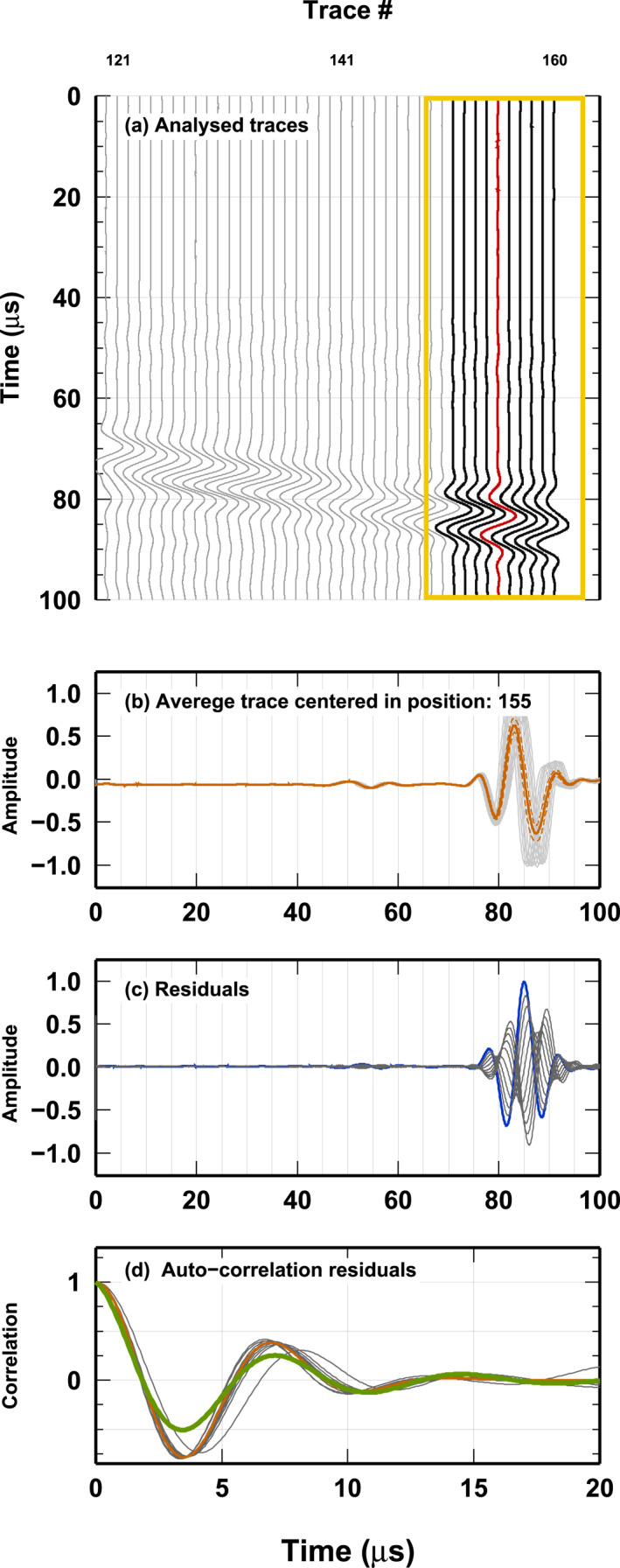
Example of data analysis for reconstructing the Covariance matrix of the error associated to trace 155. (a) Zoom of the traces close to trace 155. The yellow box indicates the traces used for estimating the standard deviation and the correlation model needed to compose the Covariance matrix. (b) Stack and standard deviation for the traces in the yellow box in (a). The orange line and the dashed orange lines represent the stack and the standard deviation, respectively. Gray lines report the traces in the yellow box in (a). (c) Residuals between the stack and each single trace in the yellow box in (a). (d) Auto‐correlation of the residuals in (c). The orange line shows the average of all autocorrelation curves (gray lines). The green line displays the best‐fitting curve, modeled using the function in Equation 6 (Kolb & Lekić, 2014).

With the assumption of independent traces, the correlation matrix **R** can be represented as a block‐diagonal matrix with *N*_*w*_ blocks, each of dimension: *N*_*s*_ × *N*_*s*_. The block **R**_*j*_ represents the error correlation within the *j*th trace and can be estimated from the data (Piana Agostinetti & Malinverno, 2018; Piana Agostinetti & Martini, 2019). However, such data‐derived correlation matrices **R**_*j*_ are often not positive definite and need to be approximated, for example, with the singular value decomposition, to use them for estimating the covariance matrix and computing the likelihood *L*(**m**_*cand*_). In this study, we make use of a correlation model that results in positive definite matrices and guarantees stable matrix inversion (Kolb & Lekić, 2014). Thus, our blocks **R**_*j*_ assume the form:

(6) $$R_{j}=R_{ik,j}=e^{-\lambda_{j}|t_{i}-t_{k}|}\cos\left(\lambda_{j}\omega_{j}|t_{i}-t_{k}|\right)$$

where *t*_*k*_ and *t*_*i*_ are the time of the *b*_*kj*_ and *b*_*ij*_ samples, respectively, while *λ*_*j*_ and *ω*_*j*_ are estimated from the data in the *j*th trace. In Figure 3, we illustrate the computation of *σ*_*ij*_, *λ*_*j*_ and *ω*_*j*_. In Figure 3a, we show how we estimate the standard deviation of each point in each trace. For the *j*th trace (red), we consider all traces between *j* − 5 and *j* + 5 and we compute a stack of these traces (Figure 3b). From the stack, we compute a residual for each trace considered (Figure 3c) and the residuals are autocorrelated. The autocorrelation functions are stacked to obtain an average autocorrelation (orange line in Figure 3d). This function is used to estimate *λ*_*j*_ and *ω*_*j*_ (green line in Figure 3d), through a 2‐parameter grid search. Our model for the autocorrelation function fits the empirical function well before 10 *μs* and somewhat over‐estimates sample correlation at longer periods, thus it should be considered a conservative model.

## 3 Exploration of the Data‐Space Through Trans‐Dimensional Sampling: Methodology

Exploring the data space of 4D seismics implies the separation of multiple sources for the “4D signal” (i.e., the signal arising when monitor and baseline surveys differ). Here we consider a simplified case using three signal sources: ambient random noise (*noise*, hereinafter), sensor misplacement (*perturbation*) and physical changes in the reservoir (*target signal*). With perfect survey repetition (no sensor misplacement) and no change in the reservoir, the unique source of 4D signal is the noise. Assuming an empirically estimated noise model, we can define our working hypothesis: in the case of a unique source of 4D signal from the noise, the fit of the monitor survey with respect to the baseline survey should close to the number of data‐points *N*_*w*_ × *N*_*s*_, where the fit is statistically represented by:

(7) $$\phi^{*}=(e_{\mathrm{ij}}^{T}\;{\left(2\times C_{\mathrm{e,ij}}^{*}\right)}^{-1}\;e_{\mathrm{ij}}),$$

which is used to compute the likelihood of the monitoring to the baseline survey:

(8) $$L^{*}=\prod_{i=1}^{N_{w}}\frac{1}{{\left[{\left(2\pi \right)}^{N_{s}}|2\times C_{\mathrm{e,ij}}^{*}|\right]}^{1/2}}\exp(-\frac{1}{2}\phi ),$$

and we assume Gaussian distributed noise with the error model defined in Section 2.1. Here, the covariance matrix $C_{\mathrm{e,ij}}^{*}$ is directly estimated from the data through their autocorrelation and their standard deviation. Here, we assume that the baseline and monitoring surveys have the same noise characteristics, however Equation 7 can be easily modified to account for different noise levels. It is interesting to note that the likelihood computation is what we need to advance our McMC sampling, following Equation 3.

When there signals in the 4D data caused by different sources, we can adopt a Hierarchical Bayes approach to define a different configuration for the covariance matrix so that the new covariance matrix will again closely fit our error model and the working hypothesis defined by Equation 8. As detailed in Bodin, Sambridge, Rawlinson, and Arroucau (2012), modifications to the covariance matrix obtained through a Hierarchical Bayes algorithm not only represent improved estimates of the data uncertainties, but also include any additional source of uncertainty arising from, for example, un‐realistic modeling or, as in our case, incorrect assumptions. In fact, the likelihood function above does represent the differences in the two surveys in case of noise only (our assumption), and the covariance matrix needs to be modified appropriately when this hypothesis is violated. In the case of sensor mis‐placement (i.e., when errors occur in the geometry of the monitor survey), the modification of the covariance matrix should be the same for all the points belonging to the misplaced traces. Conversely, when changes in the reservoir occur, the covariance matrix needs to be modified only for those seismic phases generated at the top of the reservoir for some consecutive traces (in our simplified data, from the top and the bottom in field measurements). Summarizing, we will try to define a different structure for the covariance matrix so that the modified covariance matrix will approximate our error model. In this section, we first introduce the concept of partitions of the covariance matrix and how to obtain them. Then, we illustrate what a model that represents such partitions looks like, expressing it as a vector of parameters. In the next subsection, we describe the a‐priori probability distributions of our parameters. These distributions are required in our Bayesian approach. Finally, we present the details of our “recipe” to update the McMC sampling, that is, how to choose a new candidate model from the current one.

### 3.1 Partition of the Error Covariance Matrix

Here we define a new structure of the covariance matrix as an unambiguous correspondence between a partition of the data and a partition of the covariance matrix, so that separating regions of the data space separates distortions in the covariance. Given the properties of the covariance matrix and assigning a relevant weight to each sampled point (x,t), we can create a modified covariance matrix such as

(9) $$C_{\mathrm{e,ij}}\left(m\right)=W\left(m\right)\times 2\times C_{\mathrm{e,ij}}^{*}\times W\left(m\right)$$

where

(10) $$W_{\mathrm{ij}}\left(m\right)=10^{w_{ij}\left(m\right)},$$

and *w*_*ij*_ is a weight associated to sample point (*x,t*), derived by the model sampled during the McMC process. Note that our assumptions on the original covariance matrix (block‐diagonal matrix generated from a modeled correlation function) are not necessary for generating **C**_**e,ij**_. Thus, the following discussion can be generalized to any covariance matrix. The goal now is to generate sensitive weights for all points, to be able to separate the portion of the monitor survey where the signal follows the likelihood in Equation 8, from the signal where other distortions are present. Given the nature of the distortions considered here, we can assume that, in the case of the misplacement of a single sensor, all the weights associated to the corresponding trace have to be modified by the same amount. This means that, for a given *j*, the weights *w*_*ij*_ would be the same for one entire block along the diagonal of the covariance matrix, associated to the misplaced trace. Conversely, in case of a change in the reservoir, all weights associated to the same seismic phase need to be homogeneously modified. Thus, *w*_*ij*_ would be the same for the same time interval across different traces (assuming an almost flat interface generating phases arriving almost at the same time at the receivers, as in Figure 2a at about 70 μs). This second kind of distortion strongly impacts the covariance matrix, equivalently modifying many blocks along its diagonal. Having homogeneous weights for different portions of the covariance matrix, we can create a partition of the covariance matrix based on the corresponding partition of the (*x*, *y*)‐space associated to the relevant distortion. Giving the nature of our algorithm, that is, a new way for elaborating partitions of the data, it could be categorized as a member of the family of clustering algorithms, where the number of cluster is not pre‐specified by the user or chosen during or after the data analysis, but it is self‐defined by the data themselves (e.g., Van Mechelen et al., 2018).

#### 3.1.1 Model Parameterization

**Figure 4:**
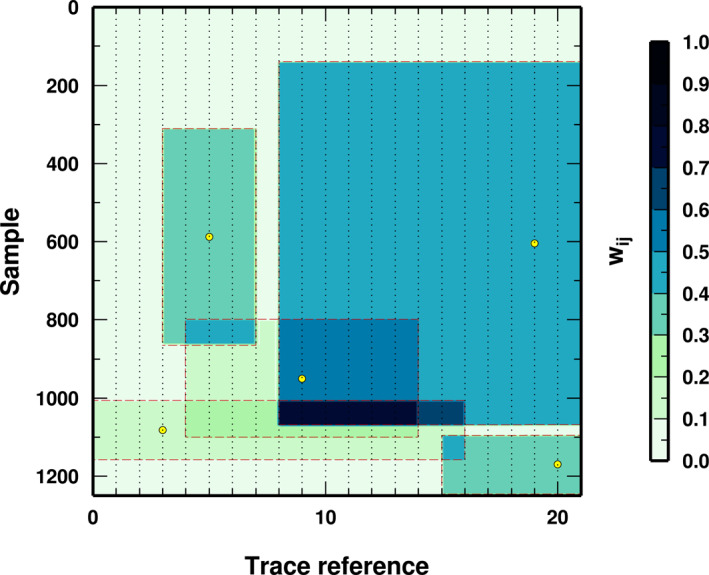
Example of a model. The rectangles represent the cell, colored according to their weights. Where cells overlap, weights are summed. Each data point (dots) has an associated weight. Data points outside all cells are associated to a weight *w*_*ij*_ = 0.0. Yellow circles represent cell nuclei. To make the figure readable, only one of every 15 data‐point is plotted.

We model our partition of the covariance matrix as rectangular partitions of the data‐space (Figure 4). Our model is represented by a variable number of rectangular patches (so‐called *cells*) that cover the data‐space, where each patch has an associated constant weight. In detail, our model **m** is composed of a scalar *n* and five *n*‐vectors, **m** = (*n*, **c**_**n**_, **r**_**n**_, **t**_**n**_, **s**_**n**_, ***π***_*n*_), where *n* is the number of cells, **c**_**n**_ the vector of position of cell centers along the *x*‐axis, **r**_**n**_ the vector of cell radii along the *x*‐axis, **t**_**n**_ the vector of the time‐position of the cell centers along the time axis, **s**_**n**_ the vector of the time‐width of the cells, and ***π***_*n*_ the vector of the cell weights. Keeping the model definition in mind, we can assume that the relevant weight for each point in the data space is the sum of the weights of the cells that extend to cover that particular point:

(11) $$w_{ij}\left(m\right)=0\;\text{if}\;\;x_{ij}\notin C_{m}\forall m=1,\ldots ,n$$

(12) $$w_{ij}\left(m\right)=\sum_{m=1}^{n}\pi_{m}\;\text{if}\;\;x_{ij}\in C_{m}$$

where $C_{m}$ represents the time‐space extension of the cell associated to the *m*th nucleus, that is:

(13) $$x_{ij}\in C_{m}\Leftrightarrow \{\begin{array}{c} c_{m}-1/2\cdot r_{m}<x_{i}<c_{m}+1/2\cdot r_{m},\\ t_{m}-1/2\cdot s_{m}<x_{j}<t_{m}+1/2\cdot s_{m} \end{array}$$

Having defined the weight for each data point as a function of the partitioning model of the data space, we now have most of the elements for sampling the model space according to our McMC strategy. In fact, the weights define the likelihood of the model from Equation 8 substituting **C**_**e,ij**_ for $C_{\mathrm{e,ij}}^{*}$, that is:

(14) $$L\left(m\right)=p\left(d|m\right)=\prod_{i=1}^{N_{w}}\frac{1}{{\left[{\left(2\pi \right)}^{N_{s}}|C_{\mathrm{e,ij}}|\right]}^{1/2}}\exp(-\frac{1}{2}\phi ),$$

where:

(15) $$\phi =(e_{\mathrm{ij}}^{T}{C_{\mathrm{e,ij}}}^{-1}e_{\mathrm{ij}}).$$

The novelty of our approach resides in the fact that, differently from standard McMC schemes, here the dependence of the likelihood function on the model is solely expressed in the covariance matrix and not in the residuals **e** (e.g., Malinverno, 2002).

Our choice of rectangular cells is optimal for the case of vertical and horizontal anomalies, because the trans‐D sampler can easily mimic this kind of distortions with a limited number of cells. However, all models sampled from the PPD will have vertical and horizontal boundaries, thus generating a somewhat “blocky” PPD. For more complex, that is, dipping, anomalies, more general functions such as anisotropic Gaussian kernels (Belhadj et al., 2018) can be adopted.

### 3.2 Priors

To make Bayesian inferences about the data partitions we define appropriate prior probability distributions on the model parameters. We make use of uniform probability distributions between minimum and maximum values for all investigated parameters. Minimum and maximum values are reported in Table 2. Uniform priors have several advantages from a computational point of view, and keep the number of pieces of prior information to a minimum (two values per parameter). We do not impose any constraints on the radius and time‐window parameters for cell centers approaching the boundary of the (*x,t*) space, that is, some cells could span outside the (*x,t*) space (this is the reason why some cells seem to have their centers not exactly in the middle of the cells in Figure 4). While this assumption can introduce some combinations of parameters with very limited impact on the likelihood function (e.g., when *c*_*m*_ is close to one or close to *N*_*W*_ and *r*_*m*_ is small), the *parsimonious* behavior of our trans‐D approach guarantees that useless cells are removed from the model at some point, thus avoiding keeping too many cells.

**Table 2:** Uniform Prior Distributions of Model Parameters in the **m** Vector

Model parameter	Minimum	Maximum
Number of cells, *n*	1	200
Cell center along *x*‐axis, *c*_*n*_	1	20
Cell radius, *r*_*n*_	1	10
Cell center along *y*‐axis, *t*_*n*_	1	1251
Cell time‐window, *s*_*n*_	1	625
Weight, *π*_*n*_	0.0	1.0

### 3.3 Candidate Selection

We now need to define how to progress in our McMC sampling, that is, how to propose a new candidate model to be compared to the current one, the so‐called *recipe*. Defining an efficient recipe, in terms of convergence to the global maximum of the likelihood function and ability to explore a (potentially) multi‐modal distribution, is fundamental for keeping the required computational resources reasonable.

Our recipe comprises seven moves, each of which represents a different way of perturbing the current model. During the definition of the candidate model only one of the moves is performed. Moves are selected with different probability. In detail, we define the following moves:

1. perturb the time‐position *t*_*n*_ of a randomly picked cell nucleus (this move has a probability of 0.15 to be selected);
2. perturb the space‐position *c*_*n*_ of a randomly picked cell nucleus (0.15);
3. perturb the time‐extension *s*_*n*_ of a randomly picked cell nucleus (0.15);
4. perturb the space‐extension *r*_*n*_ of a randomly picked cell nucleus (0.15);
5. perturb the weight *π*_*n*_ of a randomly picked cell (0.2);
6. birth of a new cell: one cell is addded to the model (0.1);
7. death of a cell: one cell is removed from the model (0.1).

Perturbation of the parameters in moves [1]–[5] are made according to the scheme in Appendix A in Piana Agostinetti and Malinverno (2010). Following this scheme, the normal proposal distributions for sampling the uniform priors have the following variances ${\sigma_{i}}^{2}$: ${\sigma_{1}}^{2}={\sigma_{3}}^{2}=8\times 10^{-3}$ for moves [1] and [3]; ${\sigma_{2}}^{2}={\sigma_{4}}^{2}=0.0025$ for moves [2] and [4]; ${\sigma_{5}}^{2}=10^{-6}$ for move [5]. Moves [6] and [7] are called trans‐dimensional moves because they imply the changing of the number of variables associated to the candidate model with respect to the current model. Such moves are defined as in Appendix B in Piana Agostinetti and Malinverno (2010), so that the determinant of their Jacobian matrix is equal to 1. We follow the approach developed in Mosegaard and Tarantola (1995) for moves [6] and [7]. Thus, we make use of a sampler that walks across the prior distributions (the so‐called *sampling from the priors* approach), and we accept or reject the candidate model with the probability in Equation 3. It is worth noting that *sampling from the priors* can be quite inefficient if the data contain a lot of information about the investigated parameters, and thus the PPD likely differs from the prior probability distribution. On the contrary, if there is limited information contained in the data, *sampling from the priors* is a convenient sampling strategy, as it removes the need to define a proposal distribution (as in, e.g., Bodin, Sambridge, Rawlinson, & Arroucau, 2012).

## 4 Results

### 4.1 Simple Cases: Misplaced Sensors or Changes in the Physical Properties of the Rocks

**Figure 5:**
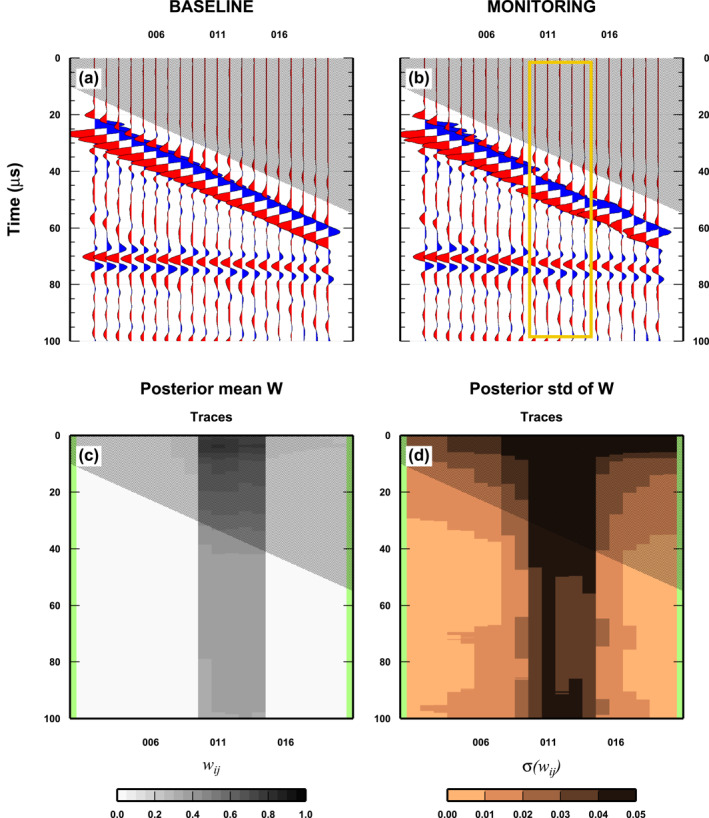
Results for a simple case: misplacement of receivers. (a) Baseline survey. The gray area denotes where the signal is absent. (b) Monitor survey. See Section 4.1 for details on how the monitor survey is created. (c) Mean posterior weight *w*_*ij*_ associated to each data point (*i*th sample on the *j*th trace). (d) Posterior standard deviation of *w*_*ij*_. The yellow box indicates the wiggles that changed between the Baseline and Monitor surveys.

In this section, we consider three simple tests. As a first illustration of the algorithm, we construct a monitor survey which mimics the mis‐placement of some sensors (Figure 5). The baseline survey is composed of 20 traces (Wiggle numbers: 5, 10, 15, …, 100) from the first experimental set‐up (Plexiglas/air). For the monitor survey, we use the same traces as in a copy of the baseline survey, and substitute five traces (Wiggle numbers: 50, 55, …, 70) with shifted traces (Wiggle numbers: 54, 59, 64,…, 74, all positions have been shifted by the same amount) from the same Plexiglas's/air experimental set‐up. In this way, the amplitude of the arrivals do not have relevant changes, but we introduce a temporal shift. It is worth noting that the number of traces used, the number of shifted traces, and the shift amplitude have been selected to keep a reasonable number of traces in the inversion (20 wiggles out of 100 available) while having enough space to introduce a significant shift in the traces (four wiggles). The results are obtained by running 5 parallel McMC samplings. Each chain is composed of 2 × 10^6^ models, half of which are discarded as part of the burn‐in phase (Somogyvari & Reich, 2019). For each chain, we used 20 CPUs on a Linux cluster for about 17 hr. Each chain has a “Master node,” which runs the McMC sampling, sends candidate models to “Slave nodes” and performs 1/20 of the Likelihood computations (i.e., one trace), and 19 “Slave nodes” which perform the remaining Likelihood computations (i.e., 19 traces). The full computation time was about 5 × 20 × 17.5 = 1,750 core‐hours. Computation time is almost constant across all tests presented in this study, due to the same number of traces and the limited number of rectangular cells used by the trans‐D sampler.

In Figure 5, we show the most relevant information extracted from the PPD, together with the monitor and baseline surveys. The misplaced traces in the monitor survey are marked (yellow box in Figure 5b). For each point in the discrete (*x*, *t*)‐space, we compute the 1D marginal PPD of *w*_*ij*_ and plot its mean posterior value (Figure 5c) and standard deviation (*std*, Figure 5d). As a rule of thumb, high values of the mean posterior *w*_*ij*_ indicate regions where the baseline and monitor surveys differ the most. Low and high values of the std differentiate well‐ and less‐constrained regions, respectively. Our results illustrate how the algorithm works in this simple case. Due to the kind of distortion used, that is, misplaced sensors, we should attribute almost the same weight to the entire set of misplaced traces. The algorithm accomplishes this task using a limited number of rectangular cells (about 20 cells, see Figure S1), confined in the vertical area of misplaced traces. The std also displays the same pattern with low values indicating a robust result. Due to the realistic nature of our test (traces obtained in laboratory and not synthetic traces), the results are not “perfect” and there are some anomalies (higher std for surface arrivals and a vertical stripe in the std plot within the misplaced traces) due to complexity in the experimental set‐up (hardware noise).

The performance of the algorithm (Figure S1) highlights some key‐aspects of the sampling. First, we are not overfitting the data because the number of cells in the sampled models is limited, and thus so is the number of inverted parameters. The acceptance probability for trans‐D moves is very low, so we need long chain (>1 million of models) to guarantee the necessary exploration of the data‐space. However, after 1 million models, the number of cells used is almost stable between 15 and 30, but not constant, that is, chains are still sampling models with variable number of dimensions but within a limited range of values.

**Figure 6:**
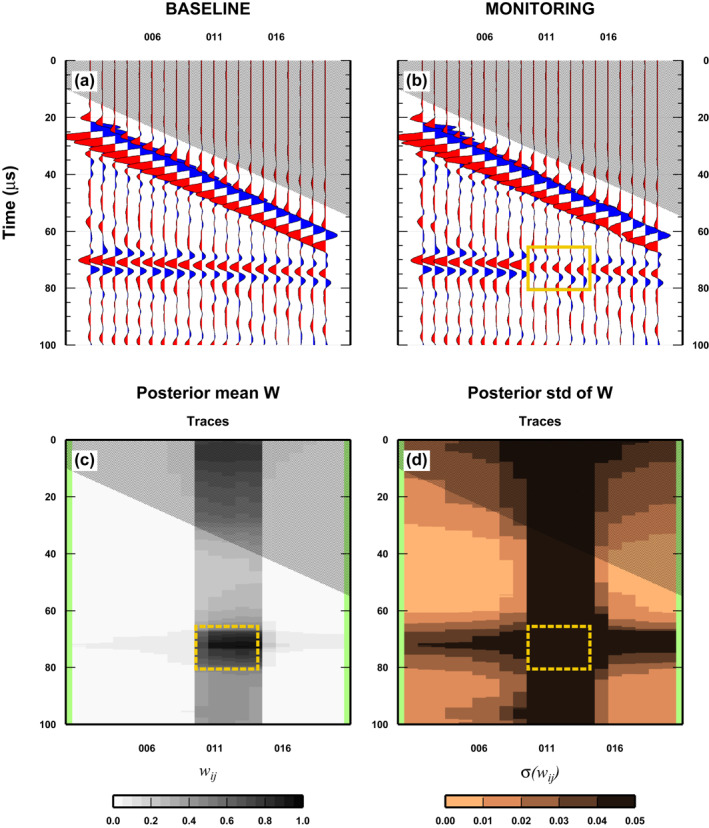
Results for a simple case: Changes in the physical properties of the reservoir. See Figure 5 for details.

Our second test is designed to complement the previous one and considers a monitor survey where only changes in the reservoir state are present (Figure 6). In this case, we make use of the same baseline as in the previous test, but in the monitor survey we substitute five traces (Wiggle number: 50 to 70) with the traces recorded at the same position but for the Plexiglas/water experimental set‐up. Both posterior mean and std of *w*_*ij*_ share the same structure, with a vertical block and a pinched horizontal structure. The main difference in the results, with respect to the previous test, is the presence of a dark (large weights) spot in the location of the change in the reservoir‐state, that is, limited to the arrivals from the top of the reservoir and not including the surface waves (Figure 6c). Also, while the results contain a vertical stripe in the mean posterior *w*_*ij*_ in the region of the reservoir changes, as in Figure 5c, the std along the same stripe is very large. Horizontally, the rectangular cells seem to be able to move slightly and the dark region in the mean posterior *w*_*ij*_ (defining the reservoir changes) propagates across some traces, suggesting that our data have a higher vertical than horizontal resolution on reservoir‐changes.

**Figure 7:**
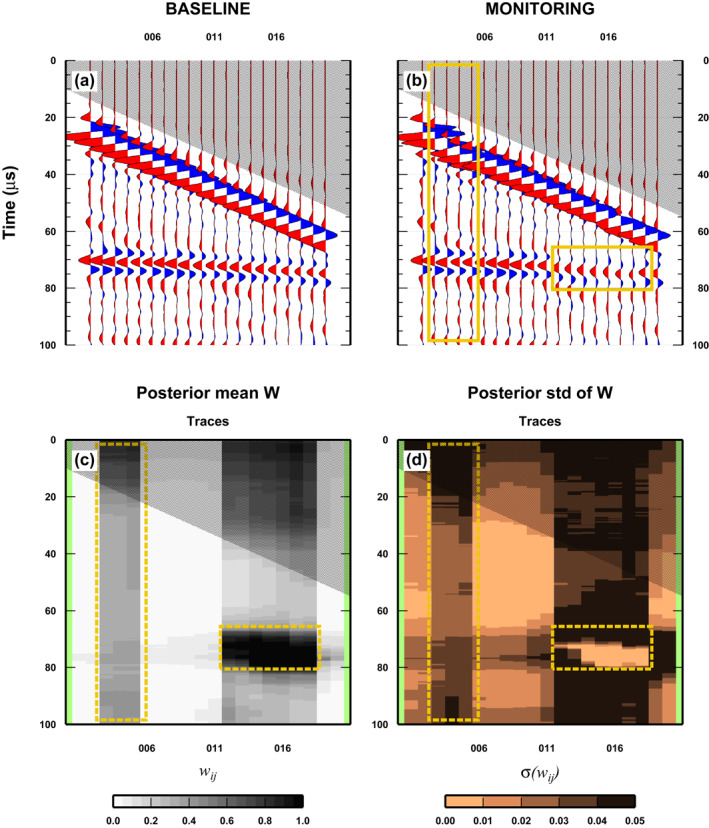
Results for a complex case: Misplacement of receivers and changes in the physical properties of the reservoir, separated. See Figure 5 for details.

The third test considers the presence of both reservoir‐changes and receiver misplacement in two separated regions of the (*x*, *t*)‐space (Figure 7). In this case, while the baseline is kept the same as in previous tests, the monitor survey is composed as follows: for the misplaced sensors, three traces (wiggle numbers 15, 20, and 25) are replaced with wiggles from the same experimental set‐up but with a four wiggle shift (so replaced with wiggle numbers: 19, 24, and 29); for the reservoir‐changes, we substitute seven traces from 60 to 90, with the wiggles recorded in the same position but with the second experimental set‐up. Note that the number of traces representing the two anomalies is different from the previous tests, to keep them separated and to be able to split it into two regions (see next section). Further analysis are needed to investigate the effect of varying the number of traces composing each anomaly on the results.

The results clearly show that, in the case of not‐interacting anomalies, the two kinds of distortions can be separately identified (Figure 7c). Both anomalies can be seen in the mean posterior of *w*_*ij*_ with the same characteristics as in the previous tests. In the analysis of the std there is a clear difference, with respect to the previous tests, in the bright spot defining the reservoir‐change, but also in the value (lower here) of the vertical stripe defining the misplaced sensors. However, such changes could be attributed to the different numbers of traces composing the anomalies (Figure 7d), indicating that the std is more sensitive to the lateral extension of the anomaly than to the mean posterior value. The bright spot in the std close to the position of the reservoir‐change resembles the “uncertainty loops” found in Galetti et al. (2015) and highlights the uncertainty in the position of the rectangular patches.

### 4.2 Complex Case: Simultaneous Retrieval of Misplaced Sensor and Changes in the Physical Properties of the Rocks

**Figure 8:**
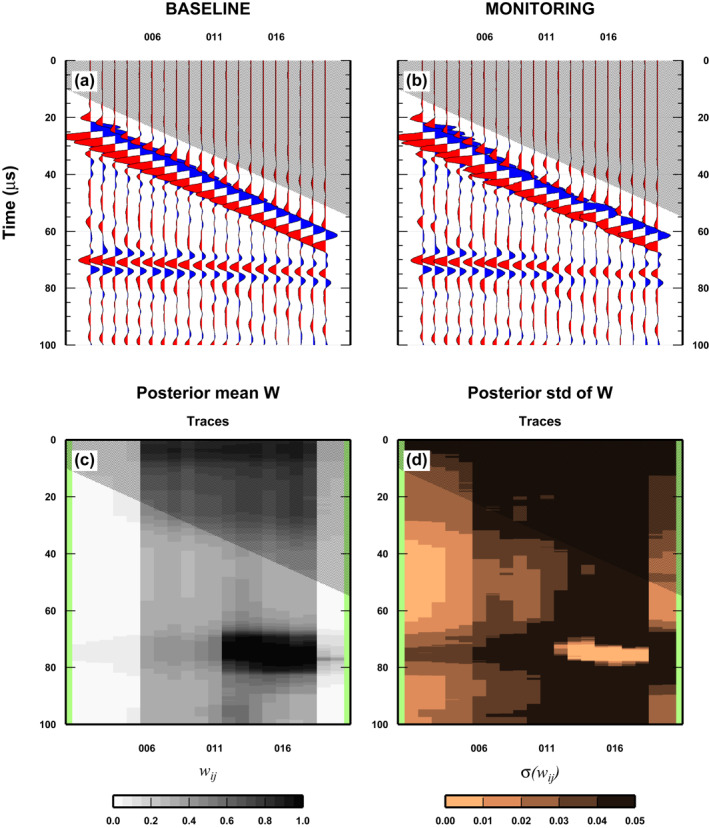
Results for a complex case: Misplacement of receivers and changes in the physical properties of the reservoir, overlapping. See Figure 5 for details. Yellow boxes indicate changes between monitoring and baseline surveys in Figure 5 have been removed for improving readability.

**Figure 9:**
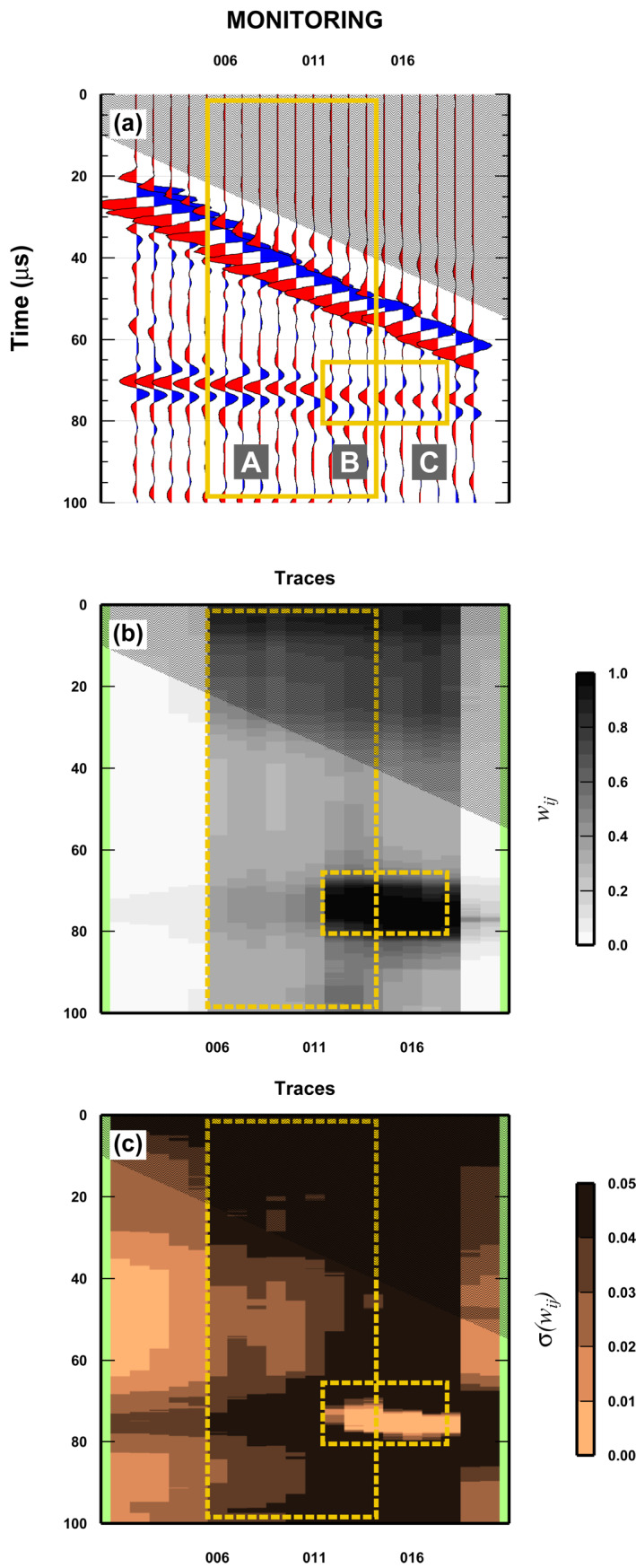
Details of the results for a complex case: Misplacement of receivers overlapping changes in the physical properties of the reservoir. (a) Monitor survey. The three letters indicate different area with [A] misplaced receivers [B] misplaced sensors and changes in the reservoir, and [C] only changes in reservoir. (b) Mean posterior weight *W*_*ij*_ associated with each data point (*i*th sample at the *j*th trace). (c) Posterior standard deviation of *W*_*ij*_. See Section 4.2 for details.

The most interesting case represents the co‐existence of both misplaced receivers and reservoir‐changes in the same region of (*x*, *t*)‐space. To test this, the baseline is kept the same as in previous tests. The monitor survey is composed of the baseline traces with substitutions in three different and contiguous regions. In the first region, called “A,” six traces are substituted by shifted wiggles from the same experimental set‐up (i.e., mimic misplacement receivers only: wiggles numbers 30, 35, …, 55 are replaced with 34, 39, …, 59). Also in the second region “B” we have misplaced traces (three traces, wiggles numbers 60, 65, and 70 replaced with 64, 69, and 74) but from the second experimental set‐up, to simultaneously reproduce both misplaced receivers and reservoir‐changes. Finally in the third region ”C,” we consider reservoir changes only. Four traces (wiggles numbers 75 to 90) are replaced with the wiggles recorded in the same position, but from the second experimental set‐up. The minimum region dimension is three traces, but the “misplaced sensors” anomaly covers nine traces, while the “reservoir‐changes” anomaly covers seven traces (Figures 8 and 9).

As expected, the outcomes from a complex case are more challenging to describe. The mean posterior of *w*_*ij*_ still clearly defines the reservoir changes as a dark (large values) elongated region that covers exactly the expected traces (Figures 8 and 9b). However, recognizing the boundaries between regions “A” and “B,” and “B” and “C” is not easy in the mean posterior. In fact, the value of the mean posterior of *w*_*ij*_ does not change significantly through regions “A” to “C” away the reservoir‐changes zone, with fluctuation given by experimental noise and lateral smearing of the reservoir‐changes anomaly. It is hard to recognize which traces have only been shifted (from the region between traces number 1 to 5 where the two surveys share the same wiggles) or which traces are both shifted and have a reservoir‐change. Knowing the monitor survey composition, we can see that more traces than the ones composing region “C” have been locally perturbed, from the occurrence of the high‐weights at localized times (dark region), but we cannot really discriminate which of the traces that also have the reservoir‐change signature have been displaced.

The results for the posterior std of the *w*_*ij*_ furnish some additional insights into the separation of the three regions. In fact, comparing both mean and std shows that the posterior std is generally uniform, but very large in the region where we only have reservoir changes (as seen also in Figure 6). The posterior std is lower and more variable for the region where we have misplaced traces (both with and without simultaneous reservoir changes). In practise, only the simultaneous analysis of both mean and std posterior for *w*_*ij*_ can somewhat unequivocally define the three regions.

Finally, the posterior std is very low in the core of the reservoir‐changes anomaly, as found in the previous test (compare to Figure 8d), likely caused by the large lateral extent of the anomaly (quite large, seven traces [one third of the total]). Moreover, we observe that the area of the std where we only have misplaced sensors is not uniform as expected, due to the interaction with the reservoir anomaly (anomaly lateral smearing). However, the std is large where the two anomalies interact.

## 5 Discussion

We propose a new methodology for exploring 4D seismic data and detecting potential noise sources other than random ambient noise, and relevant signals from the alteration of a reservoir. The algorithm has been proven to correctly perform in isolating simple case scenarios (one noise source or one reservoir change, or both present in two different portions of the 4D seismic data). In such cases, our algorithm identifies the different anomalies and their position, and it is able to characterize them in terms of both the amplitude of the posterior weights and their standard deviation. In particular, anomalous signals related to a misplacement of the sensors is identified as a broad portion of the monitoring survey where the posterior weights are uniformly increased by a limited amount, and their standard deviation is uniform too. Conversely, in the portion of the monitoring survey where the anomaly is related to a reservoir change, the posterior weights are extremely high in a localized 2D patch. Their standard deviation also displays a peculiar pattern, with very low values in the inner portion of the anomaly and very high values along its border. We suggest that the rapid change in the standard deviation is the key‐element that can define the shape of the anomaly related to reservoir changes.

In more complex cases, that is, where both noise sources and reservoir signals coexist, the interpretation of the results is more challenging. Dis‐aggregating co‐existing changes/mis‐positioning is not easy (Figure 9), but we observe that reservoir changes are always the most striking and isolated feature. Also in this case, the analysis of the standard deviation of the weights is a critical point for making inferences. In fact, even here the sharp change in the standard deviation defines the border of the anomaly given by reservoir changes. Moreover, the standard deviation also helps to define the area where the mis‐placed sensors are present (these regions have a lower standard deviation compared to area where only reservoir changes are present). It is worth noting that the estimation of the standard deviation of the weights is a brand new outcome of our algorithm, given by our statistical approach to data‐space exploration.

Our results display to some extent the boundaries of our rectangular patches (i.e., they seem to have a block‐structure). Such blockiness indicates the resolution limits of our model to some extent, and are related to our choice of rectangular partitions. In trans‐D algorithms, the effects of the parameterization on the retrieved results is an on‐going research field (e.g., Gao & Lekic, 2018). Here, we suggest that other choices of partition shape could be more efficient on bigger‐scale data, such as the *anisotropic kernels,* proposed in Belhadj et al. (2018), which could more easily reproduce the true shape of anomalies in field measurements. We anticipate that the choice of the parametrization is strictly related to the data‐space that needs to be explored. Preparing different kinds of geophysical data‐sets (see below) will probably need completely different parameterizations (e.g., a set of changepoints as in Poggiali et al., 2019). For example, a parameterization of 1D Voronoi cells (or changepoints) can be useful to create separators in data represented by time‐series of observed quantities.

**Figure 10:**
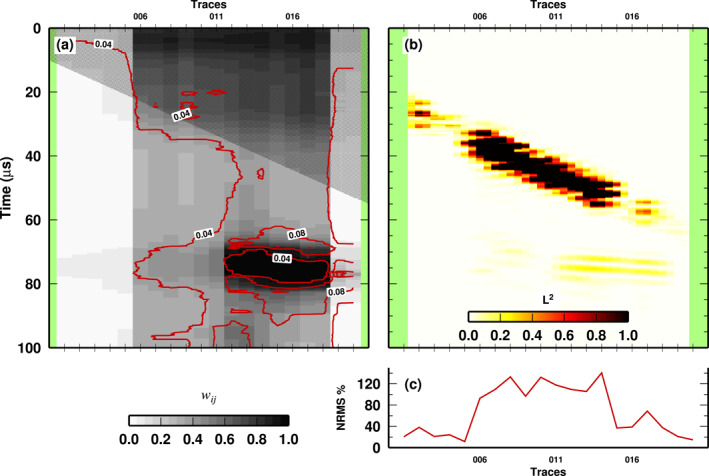
(a) Mean posterior weight *W*_*ij*_ associated with each data point (*i*th sample at the *j*th trace). Posterior standard deviation of *W*_*ij*_ is shown as red contour lines. See Section 4.2 for details. (b) Same as in Figure 2c, point‐wise L_2_ difference between monitoring and baseline surveys. (c) NRMS for each trace of the monitoring survey with respect to baseline survey. NRMS computed as in Kragh and Christie (2002).

Our approach to 4D seismic data analysis could be used to support more complex data workflows adopted in energy industries. In Figure 10, we compare the results of our complex case, with a standard analytic indicator (NRMS) commonly used in data‐workflow for 4D seismics. Comparing Figures 10a and 10b, it seems that mis‐positioning is the most impactful issue in terms of likelihood between baseline and monitoring surveys, but it is easily separated from reservoir changes, which have the strongest *W*_*ij*_ in our case. As seen in Figure 10c, NRMS is clearly higher in the area of sensor misplacement. Such an anomaly masks the signal coming from the “altered conditions in the reservoir.” In fact such a signal can be seen as a small amplitude anomaly (i.e., around 40% at trace 16–19, still higher NRMS with respect to trace 1–5 where no anomaly is present at all), but it is totally obscured between traces 11 and 15, where the dominant effect is the sensor misplacement. Our approach could be used as a support to standard data‐workflow and could save time during subsequent petro‐physical modeling of the reservoir (an extremely time‐consuming task). Because it makes no preliminary assumption on the reservoir geometry, our approach does not risk bringing an initial bias into the results and thus could furnish more reliable information on the state of the geo‐resources. As explained in Kragh and Christie (2002), the main technical risk when acquiring time‐lapse seismic data is that production‐related effects are obscured by differences incurred by a lack of repeatability of the seismic acquisition. Our approach can be used to separate the two effects. In fact, misplaced traces display a different signature in our mean posterior plots, with respect to reservoir‐changes. Moreover, the computation of the posterior std can be used to find the most promising areas where production‐related effects are statistically supported by the data.

Our novel methodology can be re‐adapted to explore different kind of geophysical datasets. In seismology, raw data are generally not used as input data to geophysical inverse problems and data preparation often occurs simply based on expert‐opinion. This is the case, for example, of the preparation of double‐difference (DD) seismic data, to improve our knowledge of the seismic velocity field in the subsurface (e.g., Qian et al., 2018). DD data are derived through the selection of paired seismic events based on some kind of distance criteria (in space and, sometimes, in time, Caló et al., 2011). Being able to prepare DD data in a more objective way could guarantee more realistic results. The preparation of DD data using a trans‐dimensional approach has been presented in Piana Agostinetti and Sgattoni (2021). Location of seismic events routinely needs seismic data preparation, where arrival times of P‐ and S‐ waves recorded by distant (from the hypothesized seismic event) seismic stations are often removed from the location workflow (or their importance is downweighted). In this case, our approach can be used in conjunction with standard location processes, so that data‐space is more rigorously explored (using a trans‐dimensional algorithm) during model‐space investigation.

## 6 Conclusions

In this study, we presented a new methodology for the exploration of the data‐space. We followed a trans‐D sampling approach to recreate and validate data‐structures in the form of partitions of the covariance matrix. We applied the new methodology to 4D seismic data acquired for monitoring the sub‐surface. Our results indicate that:

1. the trans‐D approach can be applied to data‐space exploration for defining unknown data‐structures and separating data‐volumes that are coherent with a‐priori physical hypotheses;
2. the analysis of the full PPD of the data‐structures can be used for classifying different sources of 4D signal, like repeatability noise and 4D signal from the geo‐resources;
3. In comparison with standard measures of repeatability like NRMS, our approach is less biased by the presence of different sources if 4D signal in the same data‐volume and can be used to efficiently separate such sources.

In the future, we will further develop our methodology to include different shapes and orientation of the partitions (i.e., not rectangular patches, also called *anisotropic kernels, as in* Belhadj et al., 2018) for increasing the efficiency of the McMC sampling; and to consider 3D partitions and the comparison of two entire 3D volumes.

## Supporting information

Supporting Information S1

## Data Availability

Raw data (i.e., waveforms used to compose baseline and monitoring surveys) has been archived on Mendeley Data Repository (Piana Agostinetti et al., 2021) at https://data.mendeley.com/datasets/ppdmhxf3j3/1. The Generic Mapping Tools software was used for plotting the figures of this manuscript (Wessel & Smith, 1998).
