# Supplementary material for: Exploration of Data Space Through Trans‐Dimensional Sampling: A Case Study of 4D Seismics
Source: J Geophys Res Solid Earth. 2021 Nov 26;126(12):e2021JB022343. doi: 10.1029/2021JB022343 (PMC9287047; doi:10.1029/2021JB022343)
Supplement: Supplementary file 1 — Supporting Information S1 [file JGRB-126-0-s001.pdf]

AGU PUBLICATIONS

Journal of Geophysical Research B - Solid Earth

Supporting Information for

Exploration of data space through trans-dimensional sampling: A case study of 4D seismics

Nicola Piana Agostinetti

ZED Depth Exploration Data GmbH, Vienna, Austria

Maria Kotsi

PanGeo Subsea Inc

Earth Sciences Department, Memorial University of Newfoundland, St John's

Alison Malcolm

Earth Sciences Department, Memorial University of Newfoundland, St John's

Contents of this file

Figures S1

Introduction

The supplementary Figure S1 reports the details of the inversion in the first test presented in Section 4.1. In particular, the evolution of the McMC sampling is presented in terms of the number of rectangular cells sampled along the chain, and the PPD of the number of rectangular cells. Moreover, the acceptance and rejection rates along the McMC sampling, for the moves presented in Section 3.3, are also reported.

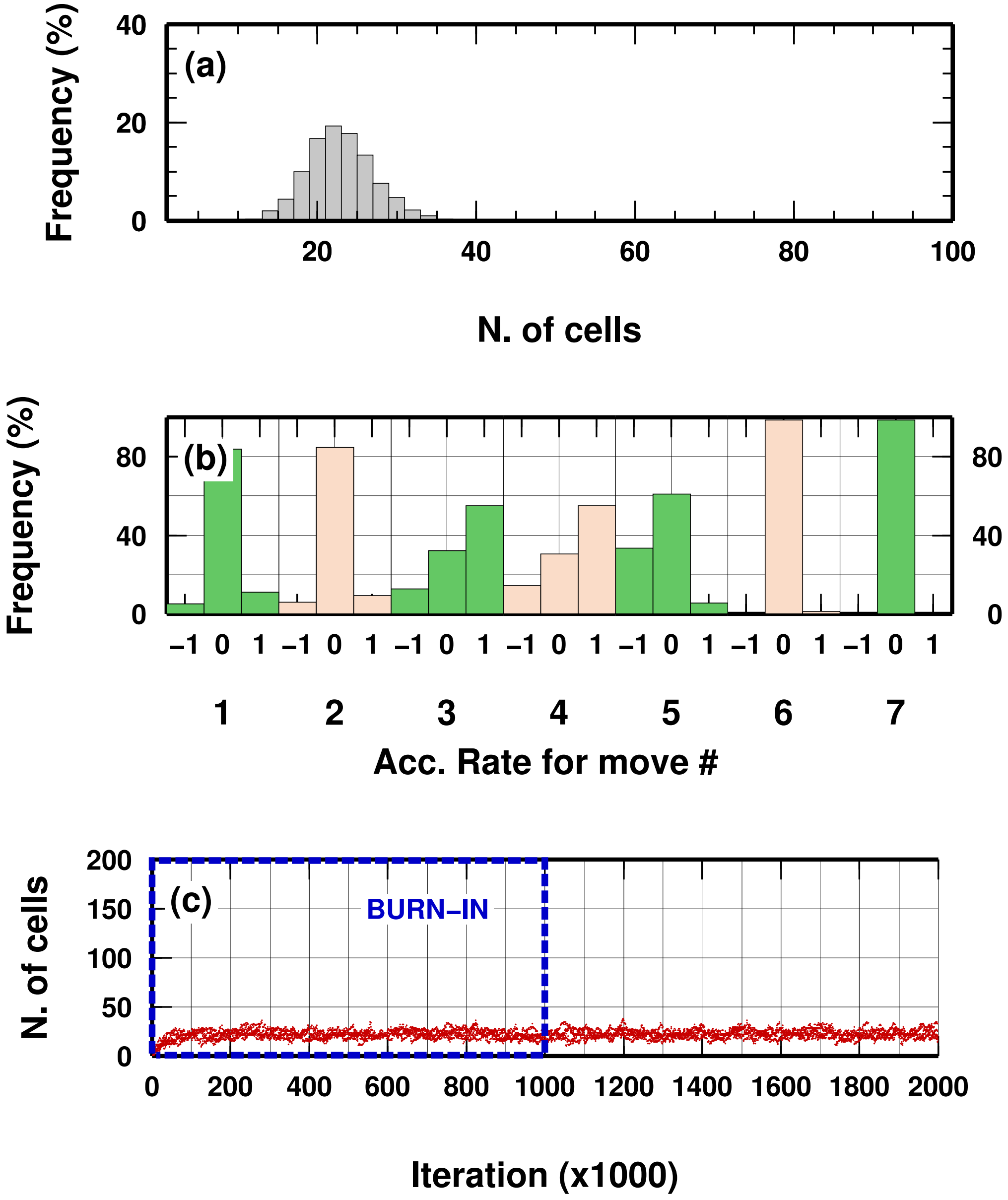

Figure S1. Details on the trans-D sampling for the simple case: misplacement of receivers. (a) PPD for the number of sectors in the model. (b) Acceptance rate for the seven moves composing the recipe for the trans-D sampling. Outcomes for each move are labelled as: “+1”, move has been accepted (candidate model improved the fit); “0”, move has been rejected; “-1”, move has been accepted, but the candidate does not improve the fit. (c) Variation of the number of cells in the sampled models for all five chains. A blue box indicates the “burn-in” period for which sampled models are not considered.
